# 

**Published:** 1914-07-25

**Authors:** 


### Gifts and Bequests.

The Royal Dental Hospital, Leicester Square, has received a legacy of £200 from the executors of the late Mr. H. S. White.

Mr. William Heaton, a retired Post Office clerk, residing in Wigan at the time of his death, has left £100 by will to the Royal Albert Infirmary in that town. He also left £1,000, out of a total estate of £2,353, to two Wigan churches, to be expended on the relief of the poor.

Mr. George Edwin Yeates, of Worcester, who left estate valued at £6,233, has bequeathed £1,000 to the Worcester Infirmary.

RATES OF SUBSCRIPTION : Twelve months, 6/6 ; Six months, 4/- ; Three months, 2/-, post free to any address in the United Kingdom. Abroad, Twelve months, 8/8 ; Six months, 5/- ; Three months, 2/6, post free.—Address The Subscription Dept., "THE HOSPITAL," The Hospital Building, 28/29 Southampton Street Strand, London W.C.

